# A revised dual-pathway model for disordered eating: A longitudinal study

**DOI:** 10.1186/2050-2974-3-S1-O56

**Published:** 2015-11-23

**Authors:** Jamie-Lee Pennesi, Tracey D Wade

**Affiliations:** 1 Flinders University, Bedford Park, Australia

## Background

To date, relatively few researchers have developed and tested integrative etiological models for the full range of disordered eating behaviours, both clinical and subclinical. In addition, much of the research conducted in this area has been correlational; few studies have investigated disordered eating growth over time. Accordingly, the current research aims to replicate and extend the dual pathway model of bulimic pathology (Stice, 2001), which has helped inform the gold standard of prevention approaches for eating disorders among adult women, to the full range of disordered eating behaviours. This research will also examine a revised model which incorporates temperament: perfectionism, self-efficacy, emotion regulation, which has shown to be predominant in the development and maintenance of eating pathology.

## Methods

Females aged 17-25 years were tested at baseline (N = 181), and at 6-month (N = 67) and 12-month follow-up (in progress). Final follow-up data for this sample will be available for analyses by the end of June 2015.

## Results

Preliminary analyses from Time 1 (baseline) suggest a promising line of enquiry with the addition of temperament (e.g., perfectionism) within the dual pathway model. Findings from Time 1, Time 2, and Time 3 will be presented.

## Conclusion

This study will add important insights to the eating disorder prevention literature and help to inform the development of approaches to prevention.

